# Bilateral Sterile Intraocular Inflammation Following Intravitreal Aflibercept 8 mg Injections: A Case Report

**DOI:** 10.3390/reports8040249

**Published:** 2025-11-28

**Authors:** Ram Cohen, Tomer Kerman, Omer Trivizki

**Affiliations:** 1 Department of Ophthalmology, Tel Aviv Sourasky Medical Center, University of Tel Aviv, Tel Aviv 64239, Israel; 2 Department of Ophthalmology, Bascom Palmer Eye Institute, University of Miami Miller School of Medicine, Miami, FL 33136, USA

**Keywords:** intraocular inflammation, aflibercept 8 mg, injections, anti VEGF, adverse events

## Abstract

**Background and Clinical Significance**: To report a case of bilateral sterile intraocular inflammation following intravitreal aflibercept 8 mg (Eylea HD) injections. **Case Presentation**: An 89-year-old woman with bilateral neovascular age-related macular degeneration (nAMD) developed blurred vision and mild ocular pain in both eyes four days after receiving aflibercept 8 mg injections in both of her eyes. Examination revealed a marked anterior chamber reaction with Descemet’s folds, 2+ vitreous cells, and 3+ vitreous haze bilaterally. Intraocular pressures were normal, and B-scan ultrasonography confirmed attached retinas with bilateral vitreous opacities. The clinical presentation initially raised concern for infectious endophthalmitis; however, the bilateral presentation, quiet conjunctivae, and prior history of sterile inflammation after aflibercept 2 mg supported a diagnosis of sterile intraocular inflammation. The patient was hospitalized and treated with intensive topical corticosteroids, antibiotics, and cycloplegics, resulting in rapid improvement and complete resolution of symptoms within four days with recovery of baseline vision. **Conclusions**: Intravitreal aflibercept 8 mg can be associated with bilateral sterile intraocular inflammation, even in patients who previously tolerated standard-dose aflibercept. Awareness of this potential adverse event is essential to avoid unnecessary interventions and to guide appropriate management.

## 1. Introduction

Intravitreal anti-vascular endothelial growth factor (anti-VEGF) therapy has revolutionized the treatment of macular diseases such as neovascular age-related macular degeneration (nAMD), diabetic macular edema (DME), and retinal vein occlusion, significantly reducing visual impairment worldwide [1]. Commonly used agents include aflibercept 2 mg, ranibizumab, and bevacizumab, which has been widely adopted as a first-line therapy for these conditions [1,2]. Although generally well tolerated, intravitreal anti-VEGF injections can occasionally cause ocular adverse events, including rare but potentially vision-threatening infectious or noninfectious intraocular inflammation (IOI) [3,4,5]. In large multicenter studies, the incidence of endophthalmitis per patient has been reported to range between 0.019% and 1.6%, while clinically significant IOI occurred in approximately 1.4–2.9% of cases [3].

In order to reduce the substantial burden of continuous anti-VEGF therapy for both patients and healthcare providers, aflibercept 8 mg (Eylea HD) was recently approved for the treatment of nAMD and DME, offering the possibility of extending treatment intervals beyond 8 weeks. Clinical trials demonstrated that visual outcomes with the 8 mg formulation administered at these extended intervals were noninferior to those achieved with the 2 mg formulation given every 8 weeks, with a comparable safety profile to the lower-dose formulation [6,7,8].

Since its introduction into clinical practice, sporadic reports and small case series have described unilateral, noninfectious IOI following aflibercept 8 mg administration, most often mild, and typically resolving with anti-inflammatory therapy [9,10,11,12,13,14,15].

In this report, we present a case of bilateral noninfectious IOI following aflibercept 8 mg injections. To our knowledge, this is the first reported occurrence with aflibercept 8 mg. Given the recent approval of this agent, documenting such an unexpected bilateral IOI provides an important addition to its emerging safety profile.

## 2. Case Report

An 89-year-old woman presented to our clinic with bilateral blurred vision and mild ocular discomfort, four days following intravitreal aflibercept 8 mg (Eylea HD) injections to both of her eyes.

Her medical history was notable for hypothyroidism treated with Euthyrox. Her ocular history included bilateral pseudophakia, glaucoma, and neovascular age-related macular degeneration (nAMD), managed with intravitreal aflibercept 2 mg at 4-week intervals. The patient presented with worsening vision in the right eye and evidence of intraretinal fluid per optical coherence tomography (OCT), whereas the left eye remained stable. Consequently, treatment in the right eye was switched to aflibercept 8 mg. The injection was performed uneventfully, with no subsequent adverse effects. Two weeks later, she was referred to the emergency department with acute vision loss in her left eye, and examination revealed a new macular hemorrhage, and therefore therapy was escalated to aflibercept 8 mg also in her left eye.

**Figure 1:**
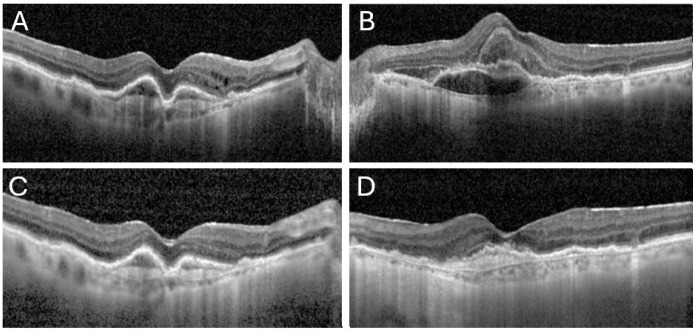
OCT images from both presentation of the patient to the ER prior to aflibercept 8 mg injections and post treatment. The first images demonstrate intra-retinal fluid and pigment epithelial detachments (PEDs) in her right eye (**A**), and sub-macular hemorrhage with subretinal fluid in her left eye (**B**). Images (**C**,**D**) show clearance of the vitreous haze in both eyes under topical treatment, with resolution of the intraretinal fluid and partial absorption of the hemorrhage in the right and left eye, respectively.

Four days following bilateral aflibercept 8 mg injections, she presented with decreased vision in both of her eyes (Figure 1).

**Figure 2:**
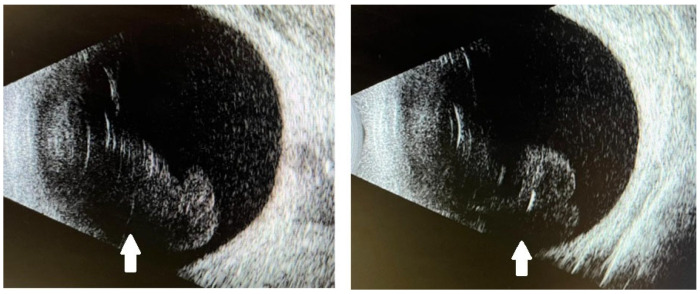
B-scan ultrasonography of both eyes showing attached retinas and symmetrical vitreous opacities (white arrows) consistent with intraocular inflammation.

On examination, best-corrected visual acuity (BCVA) had declined from 20/63 to 20/125 in the right eye and from 20/200 to 20/400 in the left eye. Slit-lamp examination revealed clear conjunctivae but demonstrated Descemet’s folds, 4+ anterior chamber (AC) cells and flare, and 2+ vitreous cells with 3+ vitreous haze in both eyes, resulting in poor fundus visualization. Intraocular pressures were within normal limits. B-scan ultrasonography confirmed attached retinas and showed symmetrical vitreous opacities (Figure 2) consistent with intraocular inflammation.

Given the acute bilateral inflammation with corneal edema and AC/vitreous involvement four days after intravitreal injections, infectious endophthalmitis was initially considered. However, the absence of conjunctival injection, the bilateral presentation, and the patient’s history of a similar sterile inflammatory reaction three weeks after aflibercept 2 mg in both eyes three years earlier favored a diagnosis of bilateral sterile intraocular inflammation. In the previous episode, hospitalization and intensive topical corticosteroid therapy had led to complete resolution within two days.

Furthermore, because the inflammation developed three weeks after administration, it was deemed unrelated to the injection, and she subsequently continued 2 mg Eylea injections without further adverse events.

**Figure 3:**
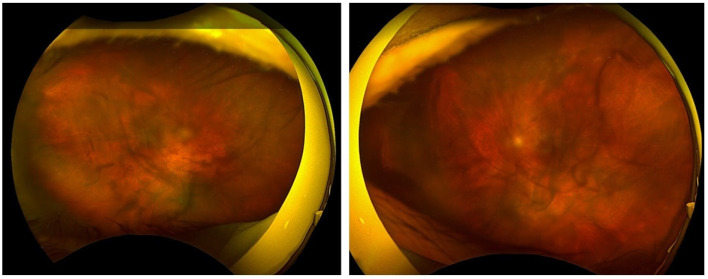
An Optos image obtained on the second day of hospitalization and treatment reveals vitreous opacities through which the posterior pole is discernible.

The patient was admitted and treated with hourly topical corticosteroids, topical antibiotics, and cycloplegics. Daily examinations and serial ultrasonography demonstrated rapid improvement, and by the second day of hospitalization and treatment the posterior segment was sufficiently visible to allow documentation using OCT (Heidelberg Engineering, Heidelberg, Germany) (Figure 1) and Optos ultra-widefield imaging (Optos^®^ 200Tx, Optos^®^, Dunfermline, U.K.) (Figure 3). Therapy was gradually tapered as inflammation subsided, and the patient was discharged when her visual acuity returned to baseline and infectious endophthalmitis was definitively excluded.

## 3. Discussion

This report describes a rare case of bilateral sterile IOI in an 89-year-old woman with nAMD, occurring four days after simultaneous bilateral intravitreal aflibercept 8 mg injections administered due to a new macular hemorrhage while on monthly aflibercept 2 mg. To our knowledge, this is the first documented case of bilateral IOI following aflibercept 8 mg administration to both eyes.

Current evidence on IOI after anti-VEGF injections indicates that bilateral presentations have been described with agents such as faricimab [16,17,18] and bevacizumab [19,20], but neither clinical trials nor published reports have documented bilateral events with aflibercept 8 mg.

Data from randomized studies have described only unilateral cases and have shown that the safety profile of aflibercept 8 mg is comparable to that of aflibercept 2 mg, for which the reported incidence of IOI per injection is very low (0.004–0.37%) [21,22,23,24]. In the CANDELA trial of patients with nAMD, one case of mild anterior chamber inflammation was observed among 44 participants, consistent with this, PULSAR trial in nAMD and the PHOTON trial in DME reported IOI in about 1% of participants. Importantly, participants in these pivotal trials were primarily treatment-naive, with no known inflammatory predispositions. In contrast, our patient was treatment-experienced and had a prior history of sterile inflammation following aflibercept 2 mg [6,7,8].

Given the recent approval of aflibercept 8 mg, several clinical reports have described IOI following its use, all of which were unilateral. Matsumoto et al. evaluated 35 eyes with nAMD and observed IOI with retinal vasculitis in three of 35 eyes (8.6%), two in treatment-naive patients and one in a previously treated patient with a history of faricimab-related IOI [9]. Binder et al. analyzed 41 patients encompassing 136 injections and identified IOI in five patients within one to three days (12.2% per patient, 3.7% per injection) [12]. Across these two series, events were unilateral, mild, and noninfectious, managed with local corticosteroids, and resolved without vision loss. In addition, several small reports have mirrored this pattern after aflibercept 8 mg, describing unilateral, mild, noninfectious IOI that responded to topical corticosteroids without vision loss [11,13,14]. By contrast, two additional reports described more severe phenotypes despite being unilateral and noninfectious: Hashiya et al. reported a case with retinal hemorrhage and vascular occlusion leading to residual visual impairment in a patient with a prior history of brolucizumab-related IOI [10], and Sisk et al. reported occlusive retinal vasculitis after aflibercept 8 mg [15]. As noted, none of these publications documented bilateral involvement.

Although the pathogenesis of sterile IOI after intravitreal anti-VEGF injections remains incompletely defined, Anderson et al. outlined three plausible mechanisms: patient-specific immune sensitization, medication-related factors, and delivery-related triggers [25]. In patient-specific immune sensitization, pre-existing or treatment-induced anti-drug antibodies (ADAs) bind the injected molecule, form immune complexes, and cause intraocular inflammation [25,26]. Medication-related factors reflect an immunogenic response to the anti-VEGF antibody itself, in which interactions of the drug’s Fc region with intraretinal Fc receptors can promote immune complex formation and inflammation [25,27]. Delivery-related triggers arise from the injection system and handling and include excipient sensitivity, silicone oil microdroplets from syringes, protein aggregates generated by agitation or freeze–thaw, and occasional preparation contamination [25,28,29,30].

In our case, the near-synchronous involvement of both eyes after bilateral injections most strongly supports patient-specific immune sensitization, with circulating ADAs encountering antigen in each eye. This interpretation is supported by the prior IOI after aflibercept 2 mg, indicating sensitization to the same molecule and a recall response on re-exposure, with the higher dose and concurrent bilateral administration likely increasing antigen exposure to a level sufficient for a clinically evident inflammatory response. Medication-related features are best considered potential amplifiers in this context, whereas delivery-related mechanisms are less likely in the absence of evidence for identical contaminant exposure in both eyes.

Notably, both our patient and a previously reported case by Hashiya et al. had a history of anti-VEG- related IOI before presenting with an atypical course, bilateral onset in our case and a more severe vasculitic phenotype in theirs [10]. These observations may indicate that prior anti-VEGF related IOI reflects immune sensitization that can manifest with broader or more severe phenotypes upon re-exposure, whether to the same molecule or to a related agent. Further study in larger cohorts is needed to validate this hypothesis.

## Data Availability

The original contributions presented in this study are included in the article. Further inquiries can be directed to the corresponding author.
